# Flexible and fine-grained simulation of speed in language processing

**DOI:** 10.3389/fpsyg.2024.1333598

**Published:** 2024-04-10

**Authors:** Xueyao Pan, Bingqian Liang, Xi Li

**Affiliations:** ^1^School of Foreign Languages and Literatures, Chongqing Normal University, Chongqing, China; ^2^School of Foreign Studies, Anhui Xinhua University, Hefei, China; ^3^Foreign Language College, Chengdu Normal University, Chengdu, China

**Keywords:** embodiment, action semantics, speed, context, mental simulation

## Abstract

According to the embodied cognition theory, language comprehension is achieved through mental simulation. This account is supported by a number of studies reporting action simulations during language comprehension. However, which details of sensory-motor experience are included in these simulations is still controversial. Here, three experiments were carried out to examine the simulation of speed in action language comprehension. Experiment 1 adopted a lexical decision task and a semantic similarity judgment task on isolated fast and slow action verbs. It has been shown that fast action verbs were processed significantly faster than slow action verbs when deep semantic processing is required. Experiment 2 and Experiment 3 investigated the contextual influence on the simulation of speed, showing that the processing of verbs, either depicting fast actions or neutral actions, would be slowed down when embedded in the slow action sentences. These experiments together demonstrate that the fine-gained information, speed, is an important part of action representation and can be simulated but may not in an automatic way. Moreover, the speed simulation is flexible and can be modulated by the context.

## 1 Introduction

How language is represented in the mind and brain has been a controversial issue. According to the symbolic view of language, concepts are represented as highly abstract and amodal symbols that are arbitrarily linked to what they represent in the real world. To understand the meaning of language is to compute these abstract symbols (Mahon, 2015; Leshinskaya and Caramazza, 2016; Machery, 2016). On the contrary, the embodied view of language argues that concepts are represented as sensorimotor information encoded in modality-specific brain areas. Language comprehension is achieved through mental simulation, that is, re-instantiating the sensory-motor states experienced in the referred events (Barsalou, 1999; Glenberg and Kaschak, 2002; Barsalou, 2016).

A large number of researches have demonstrated that action simulations occur in language comprehension. For example, Besides, Bidet-Ildei et al. (2017b) found that short-term arm immobilization affected the judgments of arm action verbs but not on the leg action verbs, which indicated that the sensorimotor simulation played an essential role in action language processing. Many neuroimaging studies provided more direct evidence by showing that the motor cortex would be activated during action verb and action sentence comprehension (Hauk and Pulvermüller, 2004; van Dam et al., 2010). Moreover, evidence from pathological studies seemed to indicate that action simulations play a causal role in action language processing. For instance, patients with movement disorders, like Parkinson’s disease and Huntington’s disease, have specific impairments in verb comprehension and production (Kargieman et al., 2014; Papeo et al., 2015; García et al., 2017). Due to the degeneration of motor areas, these patients are unable to perform action simulation, which then leads to the hindrance in action semantic understanding. These findings together suggest that language is embodied and mental simulation is necessary.

However, a much-debated question is to what extent language is embodied. In other words, to what extent do action simulations mirror the real-world actions? Some studies proposed that conceptual knowledge may be represented at multiple levels of abstraction (Binder and Desai, 2011). Accordingly, not all the aspects of the actual events could be incorporated into the simulation. On the contrary, some scholars argued that concepts are merely copies of our sensory-motor experience (Prinz, 2005). Therefore, action simulation should include every detail of the real world.

Despite the theoretical controversy, empirical studies proved that action simulations may contain many detailed aspects of an event, such as the effector (e.g., Klepp et al., 2017), the specific gesture (e.g., Liepelt et al., 2012), the strength of the action (e.g., Juarez et al., 2019), spatial information (e.g., Liu and Bergen, 2016) and so on. For example, Klepp et al. (2017) required participants to judge whether the verbs were concrete or not by making a hand or a foot response according to the geometrical shapes following the target verbs. Responses were facilitated when the effector required for the action referred to by the verb was the same as the effector specified by the geometrical shapes, which indicated that the information of effectors was simulated. Besides, Beauprez and Bidet-Ildei (2018) found that judgments of action verbs were not facilitated by the priming congruent point-light displays with modification to the kinematics applied to the main effector (e.g., the target depicted upper limb actions and the priming point-light displays had modification on the points of light representing wrists, elbows, shoulders), whereas the facilitation effect was observed when the kinematic modification was applied to body parts irrelevant to the action. These results may indicate that action simulations during language processing are somatotopic.

In a study carried out by Liepelt et al. (2012), participants were instructed to execute a hand opening or closing action in response to the color of the words “open” and “close.” Results showed that participants made faster responses when the action depicted by the word and the action specified by the color were congruent, providing evidence for the simulation of gestures. In addition, Juarez et al. (2019) proved that detailed strength information was represented in action simulations. Participants were asked to listen to action words and non-action words while holding grip force sensors. It has been found that compared to non-action words, action words elicited a bigger grip force. Moreover, Liu and Bergen (2016) showed that participants made faster semantic judgments when the direction of the action (away/toward) indicated by the sentence was congruent with the location of the button (closer to/farther from one’s body), suggesting that spatial information is part of the action simulation.

Compared with the above aspects, speed may be a more complex and abstract domain since it cannot be mapped directly into a single concrete type of experience. Understanding speed requires the integration of temporal and spatial information (Lingnau et al., 2009). Although controversial, recent studies seemed to suggest that even complex speed information is included in the mental simulation (Speed and Vigliocco, 2014; Speed and Vigliocco, 2015; Speed et al., 2017; van Dam et al., 2017). Take the study of Lindsay et al. (2013) as an example. In the eye-tracking experiment, participants listened to sentences containing verbs depicting a fast manner or a slow manner of motion (e.g., to dash vs. to dawdle) while looking at scenes describing an agent and a path that led to the goal object. It was found that participants had a shorter dwell time on the path when the verb depicted fast movement. In addition, Speed et al. (2017) proved that the simulation of speed is indispensable for the comprehension of action verbs. Healthy participants and patients with Parkinson’s disease were required to discriminate motion verbs from static verbs. It was shown that PD patients made slower judgments about fast action verbs, especially those related to hand, compared to healthy controls. Moreover, van Dam et al. (2017) unveiled that comprehending slow action sentences (e.g., *“The professor sneaked down the corridor.”*) and fast action sentences (e.g., *“The professor stormed down the corridor.”*) recruited different regions of the brain. The former elicited greater activity in the right primary motor cortex and anterior inferior parietal, while the latter led to greater activity in the right posterior superior temporal sulcus and middle occipital gyri.

However, far too little attention has been paid to whether the simulation of fine-grained information, like speed, is invariant or not. It has been theoretically assumed that mental simulation in language processing is context-sensitive rather than invariant (Barsalou, 2020). This is because words do not have invariant conceptual cores or generally valid meanings. Instead, the basic unit of word meaning is the “meaning potential” which comprises all the information that the word has been used to convey by a single individual or by the language community (Allwood, 2003; Lebois et al., 2015). Therefore, it can be inferred that the action simulations of fine-grained information may be also context-constrained.

Data from several studies provided evidence that action simulation can be modulated by the context. For example, in a study carried out by Bidet-Ildei et al. (2016), participants were instructed to identify the occurrence of congruent or incongruent point-light human biological movements after the oral presentation of action verbs embedded in either a plausible or implausible sentence (e.g., the neighbor is running in the garden vs. the garden is running in the neighbor). When the congruent action verbs were embedded in plausible sentences, the visual detection capacity and detection speed would be increased, which indicated that the plausibility of sentence context could modify the action simulation thus affecting the relationship between action verb and biological movements perception. Besides, Bidet-Ildei et al. (2017a) asked participants to listen to an action sentence depicting either a painful or painless sentence (e.g., “The boy is sitting on a chair” vs. “The boy is sitting on a nail”) and then detect point-light biological movements. Results showed that movement detection was facilitated when the sentence depicted a congruent action but only in the painless context, which indicated that the painful context hindered the motor simulation. In addition, Moody and Gennari (2010) found that sentences depicting high-effort action (e.g., “The delivery man is pushing the piano”) led to a stronger activation of the pre-motor cortex, compared to those depicting low-effort action (e.g., “The delivery man is pushing the chair”), which suggested that the simulation of strength required for comprehending the same verb could be modulated by the context. Moreover, in a study carried out by Sieksmeyer et al. (2021), hand-related or foot-related action verbs following intensifying adverbs were processed faster than those following attenuating adverbs, which could be attributed to the fact that manner adverbs modulated the amount of force involved in the action simulations.

The influence of context on action simulation becomes more complex when perspective information is taken into account, as many studies have found that the perspective adopted can also affect action simulation. For instance, van Dam and Desai (2016) asked participants to judge the sensibility of sentences denoting physical transfer either toward or away from oneself by pressing the bottom along a front-back axis or a left–right axis. Results showed that when the action direction implied in the sentences and the bottom location along the front-back axis were congruent, participants would make faster responses but only in the first-person perspective condition. However, a similar effect was also observed for third-person perspective sentences when the bottom was arranged along the left–right axis. In another study by Niccolai et al. (2021), Participants were presented with action verbs in the first-person or third-person perspective (e.g., I sing vs. He/She sings), and magnetoencephalography was measured. Compared with the third-person perspective condition, reading verbs in the first-person perspective induced stronger beta power desynchronization in the right hemisphere, especially in the lateral V5/MT+ area, the ventral posterior cingulate gyrus and posterior superior temporal sulcus, which were responsible for self-consciousness and self-other disentangling. It can be seen from these studies that different perspectives implied in the linguistic context are likely to elicit different action simulations and different neural activations.

As detailed above, while some research has been carried out on the granularity and the mediators of action simulations, these studies mainly focused on English processing and the effect of a particular modulating factor. More importantly, there have been few empirical investigations into (1) whether the language-induced simulations of fine-grained parameter like movement speed is automatic, and (2) whether the simulation of speed is context-dependent. In the present study, three experiments were set out to address these questions. Experiment 1 aims to investigate whether the detailed speed information is simulated in the processing of Chinese verbs which is a logographic writing system. It has been argued that compared to English processing, Chinese character processing may elicit increased activation in the left middle frontal gyrus (LMFG), an area adjacent to premotor regions, which may lead to increased motor demands, potentially resulting in different motor simulation during action-related language processing (Wang et al., 2019). We particularly focus on whether the speed simulation is automatic by employing a lexical decision task and semantic similarity judgment task. Experiment 2 and Experiment 3 seek to examine the context-sensitive nature of speed simulation by manipulating the linguistic information such as the prepositional phrases and pronouns in sentences.

### 1.1 Experiment 1a

This experiment aimed to investigate whether the speed information can be simulated in the processing of Chinese verbs and whether this simulation can be carried out in an automatic way by employing a lexical decision task. If the action simulations contain speed information and this fine-grained simulation is automatic, then one would expect different responses to verbs describing relatively fast or slow movement. However, if the action simulations do not include speed information or the speed simulation is task-dependent, then similar responses to different verbs should be observed.

### 1.2 Method

#### 1.2.1 Participants

35 native Mandarin speakers who grew up in Mainland China (10 males and 25 females, mean age = 21.74 years, SD = 1.27) took part in this experiment. All participants were undergraduate students recruited from Anhui Xinhua University, with normal or corrected-to-normal vision. They were asked to provide written informed consents before participation and got paid after the experiments.

#### 1.2.2 Materials

32 fast verbs (e.g., “奔跑,” to run) and 32 slow verbs (e.g., “漫步,” to stroll) were used in this experiment. Half of the fast verbs depicted full body action (e.g., “奔逃,” to flee) and the other half described hand/arm movements (e.g., “鞭打,” to whip). Similarly, half of the slow verbs were full-body verbs (e.g., “缓行,” to amble) and the other half were hand/arm verbs (e.g., “抚摸,” to stroke). The reason for distinguishing between full-body verbs and hand/arm verbs in both fast and slow verbs is that these two types of verbs may have different levels of sensorimotor representations, since the whole-body may be related to the larger representation in the sensorimotor cortex than the hand (Bidet-Ildei et al., 2017b). In addition, 64 pseudo-words were created which can be divided into two groups. Half of the pseudo-words are formed by retaining the first character of each real word (e.g., fast/slow, full body/hand verbs) and replacing the second character with another character sharing the same stroke count to create a nonsense two-character combination. The other half of the pseudo-words are formed by retaining the second character of each real word and substituting the first character with another one. It’s important to note that the resulting two-character nonwords do not occur in actual language usage.

Verbs were rated by another 20 Mandarin speakers who did not participate in the formal experiment in terms of speed on a 7-point scale questionnaire ranging from 1 (being very slow) to 7 (being very fast). The averaged speed rating for fast verbs was 4.78 (SD = 0.51, range = 4.1–5.8; fast full-body verbs *M* = 4.95, SD = 0.52, range = 4.1–5.8; fast hand verbs *M* = 4.61, SD = 0.44, range = 4.15–5.4), and the averaged speed rating for slow verbs were 2.78 (SD = 0.34, range = 2.15–3.4; slow full-body verbs *M* = 2.83, SD = 0.31, range = 2.15–3.2; slow hand verbs *M* = 2.73, SD = 0.36, range = 2.15–3.4). The difference in speed rating across different conditions was significant (*F* (3, 60) =125.09, *p* < 0.001, *η*^2^ = 0.862). However, rating scores of fast full-body verbs and that of fast hand verbs had no significant difference (*p* = 0.13), and rating scores of slow full-body verbs and that of slow hand verbs did not differ significantly (*p* = 1.00).

Meanwhile, verbs were rated in terms of the extent to which the hands/arms, feet/legs and torso are involved in the action on a 7-point scale questionnaire (1 being low involvement and 7 being high involvement). Full-body verbs had a higher rating score on the involvement of feet/legs (*t* = 23.92, *p* = 0.00), and the involvement of the torso (*t* = 5.13, *p* < 0.001), while the hand verbs had a higher rating score on the involvement of hands/arms (*t* = 18.24, *p* < 0.001), which justified our classification of full-body verbs and hand verbs. In addition, the effort of action implied by verbs was also rated. Fast verbs had higher effort ratings compared with slow verbs (*t* = 12.5, *p* < 0.001). However, full-body verbs and hand-verbs did not differ significantly neither in fast condition (*t* = 0.11, *p* = 0.91), nor in the slow condition (*t* = 1.16, *p* = 0.27). Besides, verbs were matched in terms of frequency based on the corpus of the Center for Chinese Linguistics at Peking University (*F* (3, 60) =0.974, *p* = 0.411, *η*^2^ = 0.046), and strokes (*F* (3, 60) =0.893, *p* = 0.450, *η*^2^ = 0.043) (see Table 1 for details).

**Table 1:** The psycholinguistic variables for all experimental conditions.

	Fast hand verbs	Slow hand verbs	Fast full-body verbs	Slow full-body verbs
Frequency	617.25 (610.22)	765.94 (964.32)	1091.23 (1063.4)	608.06 (955.27)
Stokes	16.69 (5.15)	18.63 (2.92)	18.56 (5.9)	19.13 (3.54)
Speed rating	4.61 (0.44)	2.72 (0.36)	4.95 (0.52)	2.83 (0.31)
Hands involvement	5.77 (0.18)	4.87 (0.24)	4.11 (0.35)	2.14 (0.26)
Feet involvement	2.66 (0.5)	2.46 (0.71)	5.4 (0.41)	4.85 (0.15)
Torso involvement	3.77 (0.51)	3.29 (0.63)	4.99 (0.37)	3.52 (0.26)
Effort rating	4.83 (0.64)	3.03 (0.77)	4.81 (0.51)	2.8 (0.28)

#### 1.2.3 Procedure

Participants were seated in a comfortable chair approximately 50 cm from a computer. Each trial began with a fixation “+” displayed at the center of the screen for 500 ms in order to draw the participants’ attention. Then, verbs or non-words were presented for 1,500 ms. Participants were instructed to judge whether the target was a real word or not by pressing one of two keys as quickly and accurately when they saw the target. The key configuration was counterbalanced across participants so that for half of them, the “F” key indicated a “YES” response and “J” key provided a “NO” response, while the other half followed the reverse configuration. The intertrial interval varied randomly from 300 to 500 ms. Both words or non-words were presented in font Song, 32 points, black-on-white and in the center of the screen. All experiments were controlled by the software E-Prime 2.0 (Psychology Software Tools, Inc.).

### 1.3 Data analysis

Reaction times of incorrect responses and the extreme data beyond 2.5SD from the mean RT of each condition were excluded from data analysis, with less than 2.03% of total trials being rejected. The mean reaction times to target in each condition are summarized in Table 2.

**Table 2:** Average reaction times (RTs) with standard derivations (SDs) in Experiment 1a.

	Fast-peed (ms)	Slow (ms)
Full-body	592 ± 125	611 ± 136
Hand	599 ± 126	610 ± 130

Linear mixed model analyses on log-transformed RTs were used with R program (Version 4.02: R Core Team, 2016). The lme4 (version 1.1–13, Bates et al., 2015) and lmerTest (Kuznetsova et al., 2017) packages were used. The backward elimination and log-likelihood tests were used to evaluate the models. In the initial models, the fixed factors included: the *speed* (fast vs. slow) and *effector* (hand vs. full-body) implied by the target. And the interaction between *speed* and *effector* was also included in the initial models. In addition, participants and items were included as random factors. We started with a maximal random-effects structure and simplified the model in cases of convergence failure, which led to the inclusion of *speed* and *effector* as a by-participant random slope. The ANOVA function was then used to calculate the significance of fixed factors.

## 2 Results and discussion

According to the best-fit model, the verb recognition was somewhat influenced by *speed* (*β* = 0.02, SE = 0.01, *t* = 1.96, *p* = 0.06). The main effect of *speed* was marginally significant (*x^2^*(1) = 3.80, *p* = 0.05), but the main effect of the *effector* and the interaction effect between *speed* and *effector* were insignificant (*ps* >0.10). Further analysis showed that the reaction time for the fast verbs was marginally significantly shorter than that for the slow verbs (*β* = −0.015, SE = 0.008, *t* = −1.958, *p* = 0.055).

Although there was a trend that fast verbs were recognized relatively faster than slow verbs, the difference was not significant after all. One possible explanation for this might be that the speed information is not part of the mental simulation. This account must be approached with some caution because previous studies seem to indicate that simulation of speed is an involved component in the comprehension of language about speed (e.g., Speed and Vigliocco, 2014; Speed et al., 2017). Another possible explanation for this is that speed information was not automatically and comprehensively simulated due to the nature of the task used here. According to the embodied view of language comprehension, multimodal simulation makes a great contribution to understanding the semantics of language (Barsalou, 1999, 2008, 2020). Since simulation mirrors, at least to some extent, the real-world sensory-motor experience, lexical semantic content can be grounded in the experiential traces stored in sensorimotor brain regions (Glenberg, 1997; Barsalou, 2008). However, it has been suggested that the intensity of simulation may be modulated by the tasks (e.g., Barsalou, 2008; Louwerse and Jeuniaux, 2008; Louwerse and Jeuniaux, 2010; Louwerse, 2011). For example, Louwerse and Jeuniaux (2008) holds that both embodied simulation and linguistic factors (e.g., the order in which words appear) contribute to conceptual processing. Linguistic factors play a leading role in shallow cognitive tasks to form rough but good-enough representations, while embodied simulation dominates in deeper cognitive tasks to form a full-fledged situation model. In this experiment, fast verb processing should have required the simulation of fast-speed movements and led to the activation of brain areas encoding fast movement, resulting in a significantly faster action response. However, the mental simulations take time to develop (Barsalou et al., 2008). The time-pressured lexical decision tasks may not require deep semantic processing and can be completed through linguistic information like word co-occurrence (Louwerse and Jeuniaux, 2008). In other words, comprehensive mental simulations may not be required in this experiment. Therefore, speed information was not comprehensively simulated, which prevented differences in reaction times between these two types of verbs.

### 2.1 Experiment 1b

Experiment 1a provided evidence suggesting that speed simulation might not be automatic but rather task-dependent. Specifically, in shallow semantic processing tasks, the simulation of speed information may not be necessary, whereas in deep semantic processing tasks, it is more likely to be engaged. In light of this, Experiment 1b was conducted using a semantic similarity judgment task that requires explicit and deep semantic processing to further explore whether speed information is part of the mental simulation.

### 2.2 Method

#### 2.2.1 Participants

30 participants who participated in Experiment 1a also took part in Experiment 1b (8 males and 12 females, mean age = 21.93, SD = 1.28).

#### 2.2.2 Materials

The 32 fast verbs and 32 slow verbs used in Experiment 1 were also used in Experiment 2. In addition, two sets of 32 verbs of no movement (e.g., “站立,” to stand) were used in the experiment. The speed, involvement of hands/arms, feet/legs and torso, and the effort implied by these relatively static verbs were also rated on a 7-point scale questionnaire by the same raters in experiment 1. The averaged speed rating and effort rating of relatively static verbs were 1.73 (SD = 0.15, range = 1.5) and 2.01 (SD = 0.28) respectively and were significantly different from speed verbs. Besides, these relatively static verbs did not significantly differ from the speed verbs in terms of involvement of hands/arms (*M* = 1.95, SD = 0.43; *p* = 0.23) and feet/legs (*M* = 2.69, SD = 0.97; *p* = 0.94), but they are significantly different in the involvement of torso (*M* = 2.95, SD = 0.62; *p* < 0.01). Fast verbs, slow verbs and relatively static verbs were matched in terms of frequency (*F* (2, 125) = 0.29, *p* = 0.75) and strokes (*F* (2, 125) = 1.16, *p* = 0.32). Speed verbs and relatively static verbs were divided into two sets to serve as separate blocks within the experiment. In one block, participants should distinguish fast actions from static actions, while in another block, they had to distinguish slow actions from static actions. Each block contained 64 triplets with each item serving as the target, match and filler once.

#### 2.2.3 Procedure

Participants were seated in a comfortable chair approximately 50 cm from a computer. For each trial, a fixation “+” was presented at first for 500 ms to draw the participants’ attention. Then, three verbs were represented in the form of a triangle. Participants were instructed to judge which of the two bottom words was most similar in meaning to the top one by pressing “F” key (indicating the left one) or “J” key (indicating the right one) as quickly and accurately as possible. The stimuli stayed on the screen until the participant made a response or the trial had timed out (after 2,500 ms). In the practice session, participants were told that verbs would be similar in describing movement, and feedback was given on each trial.

### 2.3 Data analysis

All incorrect responses and responses beyond 2.5SD from the mean RT were not included in the data analysis, with less than 3.5% of total trials being rejected. The results are summarized in Table 3. The log-transformed reaction times were analyzed using linear mixed modeling. Similar to Experiment 1a, *speed* and *effector* and their interaction served as fixed-effect predictors and the participants and items as a crossed-random factor. According to the best-fit model, the *effector* was included as a by-participant random slope.

**Table 3:** Average reaction times (RTs) with standard derivations (SDs) in Experiment 1b.

	Fast-peed (ms)	Slow (ms)
Full-body	1,182 ± 335	1,264 ± 395
Hand	1,288 ± 400	1,372 ± 446

## 3 Results and discussion

**Figure 1:**
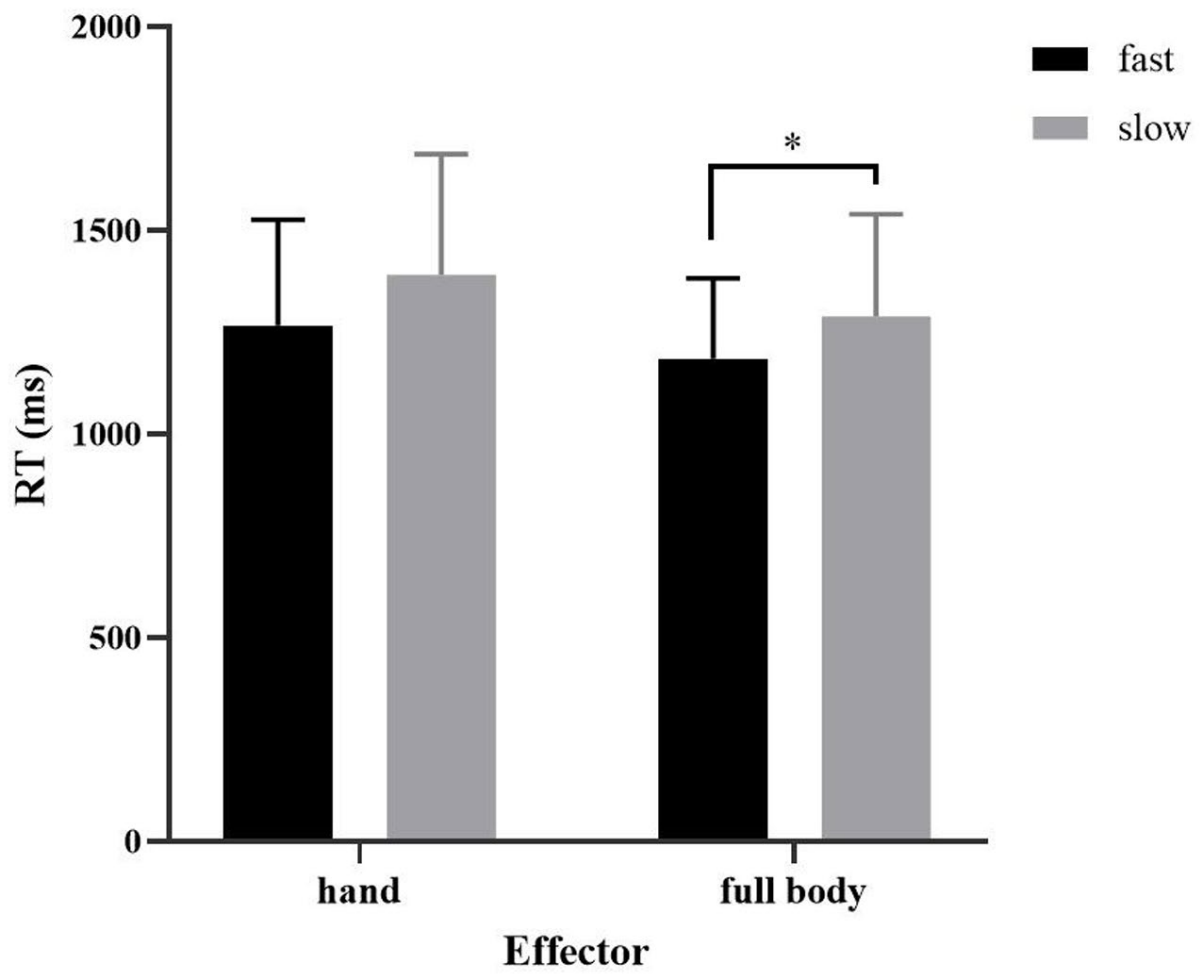
The mean reaction time (ms) in Experiment 1b. Asterisks indicate significant difference at *p* < 0.05. Error bars represent one standard error.

The reaction time was significantly influenced by both *speed* (*β* = 0.04, SE = 0.01, *t* = 4.39, *p* < 0.001) and *effector* (*β* = 0.03, SE = 0.01, *t* = 3.18, *p* < 0.001) (see Table 4 and Figure 1). The main effects of *speed* and *effector* were significant but the interaction effect between these two factors was not significant (*x^2^*(1) = 0.19, *p* = 0.66). It has been found that the fast verbs were recognized significantly faster than slow verbs (*β* = −0.04, SE = 0.01, *t* = −4.39, *p* < 0.001). And the full-body verbs were judged significantly faster than hand verbs (*β* = −0.03, SE = 0.01, *t* = −3.19, *p* < 0.001). To be more specific, the reaction times for fast full-body verbs were faster than that for slow full-body verbs (*β* = −0.03, SE = 0.01, *t* = −2.81, *p* = 0.01), and the reaction times for fast hand verbs were faster than that for slow hand verbs (*β* = −0.04, SE = 0.01, *t* = −3.40, *p* < 0.001). In addition, the responses to fast full-body verbs were faster than those to fast hand verbs (*β* = −0.03, SE = 0.01, *t* = −2.04, *p* = 0.04), and the response times for slow full-body verbs were faster than that for slow hand verbs speed (*β* = −0.03, SE = 0.01, *t* = −2.61, *p* = 0.01).

**Table 4:** Experiment 1b, RT data: model output from the best-fit linear mixed-effect model.

Random effects	Name	Variance	SD	Correlation
items	Intercept	0.0008	0.0292	
participants	Intercept	0.0071	0.0841	
	effector	0.0003	0.0184	0.59
Residual		0.0091	0.095	
Fixed effects	Estimate	SE	*t*-value	*p*-value
Intercept	3.0898	0.0159	193.85	<0.001
speed	0.0376	0.0086	4.39	<0.001
effector	0.0293	0.0092	3.18	0.002
speed: effector	0.0075	0.0171	0.44	0.66

These results are partially consistent with that of Speed et al. (2017) who found that the judgment of slow action verbs took longer than that of fast action verbs by using a similar semantic similarity judgment task. When distinguishing fast or slow action verbs from static verbs, participants had to make a detailed semantic analysis of verbs, thus the speed information had to be simulated in depth. Since fast verb comprehension and slow verb comprehension required the simulation of fast action and slow action respectively, the corresponding brain areas encoding different speed information were activated which then led to the different action responses. Therefore, these findings further support the idea that speed information is encoded in the mental simulation of action and suggest that the intensity of this fine-grained simulation can be modulated by tasks.

It’s interesting to see that the full-body verbs were judged faster than hand verbs. A possible explanation for this might be the interaction between the motor system and the mental simulation of actions. According to the Hand-Action-Network Dynamic Language Embodiment (HANDLE) model (García and Ibáñez, 2016), the motor-language coupling involves three interconnected and overlapped neural networks, that is, the hand motor network responsible for action execution and embodied semantic processing, the non-motor semantic network and the lexical-level network. When participants are instructed to make hand responses after the presentation of hand verbs, they would make unconscious motor preparation at first, which primes the activation of the hand motor network. Then, the presentation of hand verbs would lead to the supra-threshold activation in the lexical network due to the orthographic processing and in the hand motor network due to the embodied simulation. Within a certain period of time, the supra-threshold activation level in the hand motor network would be maintained, which indicates that the hand motor network is fully engaged and cannot immediately participate in hand action implementation, thus the hand response would be interfered with. The relatively slow hand verb judgment in this experiment may also be attributed to the motor-language coupling. Since the motor circuits were engaged in the simulation of hand actions in service of hand verb comprehension, the button pressing was disturbed and slowed down. In contrast, the full-body verb comprehension did not recruit the hand motor network, thus the hand network can be fully engaged in hand action execution, resulting in fast bottom pressing.

### 3.1 Experiment 2

Experiment 1 supports the idea that speed information can be simulated in action verb processing, though it may not necessarily be done automatically. However, no experiment to date has specifically examined whether the simulation of speed is stable. This experiment tested the alternative possibilities that mental simulations during verb comprehension spontaneously elicit the simulation of speed in a default way regardless of context and that simulation of speed might be modulated by context. It is worth noting that some isolated verbs like *dash* may emphasize specific speed information (e.g., to move very quickly), which usually leads to fast action simulation. However, these verbs can be embedded in sentences either describing fast movements or describing slow movements. If the speed information is simulated in an invariant and default way, then one may expect similar responses to verbs embedding in different sentences. However, if the speed simulation is flexible, verb recognition in different contexts would be different.

### 3.2 Method

#### 3.2.1 Participants

Another 30 native Mandarin speakers with normal or corrected-to-normal vision were recruited from Anhui Xinhua University (10 males and 20 females, mean age = 22.1 years, SD = 1.32). Participants were informed about the experimental procedures and signed informed consent forms before the experiment. Participants received some gifts for their participation.

#### 3.2.2 Materials

The critical stimuli comprised 120 sentences. All experimental sentences were of the form “Pronouns Prepositional-Phrases Verb” describing either a relatively fast or a relatively slow event and from either a first-person, second-person or third-person perspective. All the sentences contained fast verbs (e.g., “飞奔,” to dart) (mean speed rating scores = 5.2, SD = 0.69) but differed in terms of the context. By changing the prepositional phrases, we created relatively fast (e.g., “在操场上飞奔,” to dart on the playground) and relatively slow context (e.g., “在陡坡上飞奔,” to dart on the steep slope). All complements were matched in terms of strokes (*mean fast action sentences* = 16.55, *mean slow action sentences* = 17.40; *t* = −6.15; *p* = 0.55) and frequency (*mean fast action sentences* = 2378.60, *mean slow action sentences* = 1673.55; *t* = 1.66; *p* = 0.11) according to the corpus of the Center for Chinese Linguistics at Peking University. Then each sentence, with either fast or slow context, was described from a first-person, second-person or third-person perspective by using the pronouns I, You or He/She. Therefore, the critical sentences belonged to one of six experimental conditions (20 sentences per condition): (1) sentences denoting fast events from the first-person perspective; (2) sentences denoting slow events from the first-person perspective; (3) sentences denoting fast events from the second-person perspective; (4) sentences denoting slow events from the second-person perspective; (5) sentences denoting fast events from the third-person perspective; (6) sentences denoting slow events from the third-person perspective.

Each sentence was rated in terms of the speed implied in the sentences and the semantic plausibility by 20 participants who did not participate in the main experiment on a scale from 1 (being very slow; completely implausible in semantics) to 7 (being very fast; completely plausible in semantics). The speed rating of fast action sentences (*M* = 5.04, SD = 0.76) was significantly different from slow action sentences (*M* = 4.13, SD = 0.44) (*t* = 4.18, *p* < 0.001). And the fast action sentences (*M* = 5.37, SD = 0.56) were more semantically plausible than slow action sentences (*M* = 4.21, SD = 0.71) (*t* = 4.71, *p* < 0.001).

In addition, 120 filler sentences were created by changing the final words of the critical sentences. The final words in filler sentences were pseudowords devoid of meaning, which were constructed by changing one character of the critical verbs.

#### 3.2.3 Procedure

Each trial began with the presentation of a fixation “+” for 500 ms, followed by an incomplete sentence without the final word. After 1700 ms the text was replaced with a blank screen lasting for 300 ms. Then the final word/pseudoword was displayed for 1,500 ms followed by an intertrial interval varied randomly from 300 to 500 ms. Once the final word/pseudoword was presented, participants had to make a lexical decision as quickly and accurately as possible by pressing “F” key for the real words or “J” key for the pseudowords. The key configuration was counterbalanced across participants. Both the sentence stems and the final words/ pseudowords were displayed in font Song with a size of 32 points and in black against a white background. All experiments were controlled by the software E-Prime 2.0 (Psychology Software Tools, Inc.)

### 3.3 Data analysis

Only correct responses and reaction times within the 2.5SD from the mean RT were included in the data analysis. The results across conditions are summarized in Table 5. The linear mixed effect models were used to analyze the log-transformed reaction times. In the models, fixed-effect predictors included *context* (fast action vs. slow action), *perspective* (first-person vs. second-person vs. third-person), and *sentence plausibility* was deemed as a covariate to control for its potential influence. The participants and items were crossed-random factors, but with no random slope in the best-fit model.

**Table 5:** Average reaction times (RTs) with standard Derivations (SDs) in Experiment 2.

	Fast-action (ms)	Slow-action (ms)
First-person perspective	598 ± 121	621 ± 128
Second-person perspective	623 ± 126	618 ± 118
Third-person perspective	617 ± 126	611 ± 116

## 4 Results and discussion

**Figure 2:**
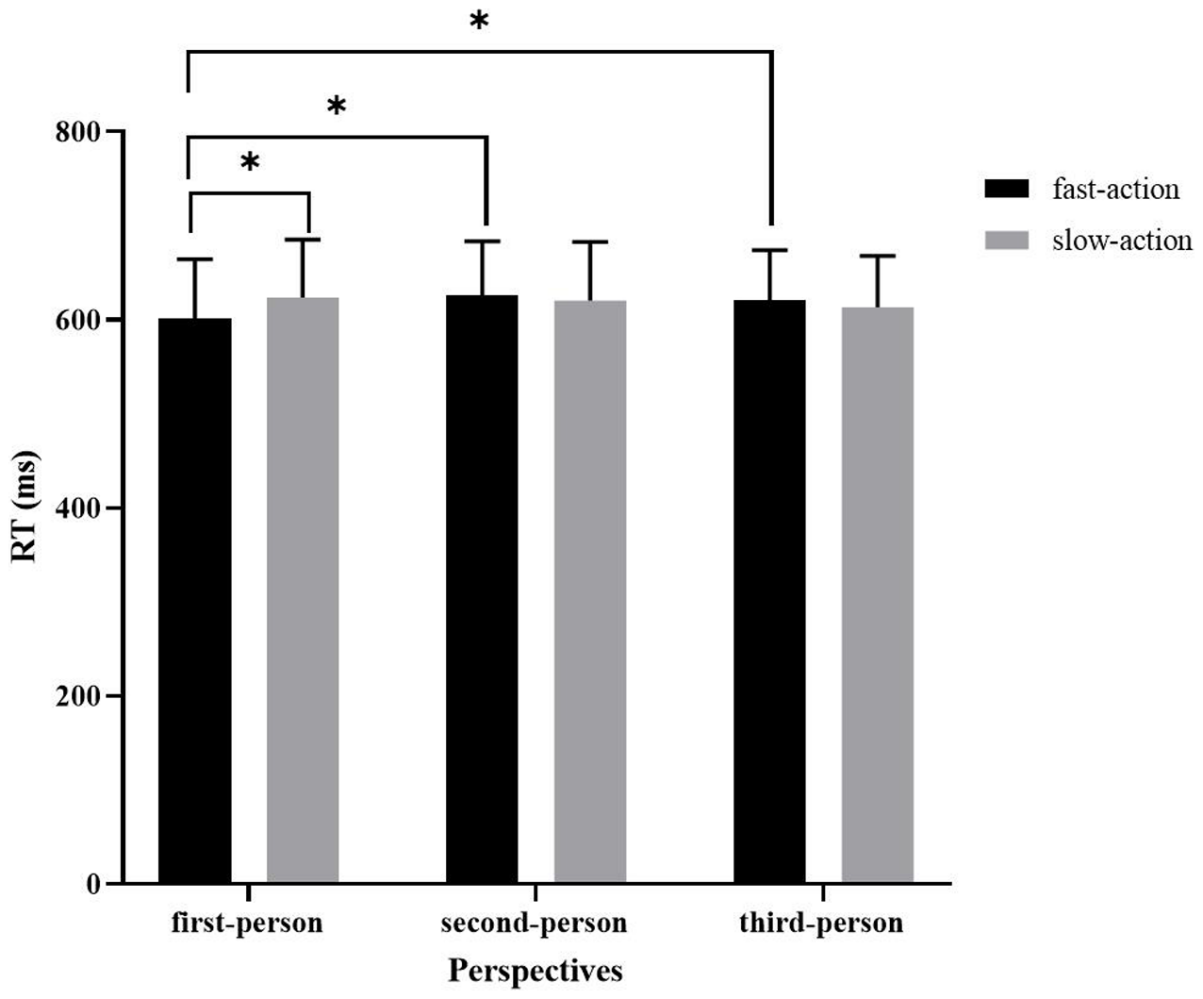
The mean reaction time (ms) in Experiment 2. Asterisks indicate significant difference at *p* < 0.05. Error bars represent one standard error.

The significant main effect of *perspective* was observed (*x^2^*(2) = 6.54, *p* = 0.04) as well as the interaction effect of *perspective* and *context* (*x^2^*(2) = 9.82, *p* = 0.01) (see Table 6). Further analysis showed that verbs in first-person perspective fast action sentences were recognized faster than those in first-person perspective slow action sentences (*β* = −0.02, SE = 0.01, *t* = −2.61, *p* = 0.01). However, in the second-person and third-person perspective conditions, the fast action sentences did not significantly facilitate verb recognition ( *ps*> 0.10). Moreover, in the fast action condition, sentences depicted from the first-person perspective significantly facilitated verb recognition compared with those depicted from the second-person perspective (*β* = −0.02, SE = 0.005, *t* = −3.78, *p* < 0.001) and third-person perspective (*β* = −0.01, SE = 0.01, *t* = −2.72, *p* = 0.02) (see Figure 2). However, in the slow action condition, perspective did not significantly affect verb recognition.

**Table 6:** Experiment 2 RT data: model output from the best-fit mixed-effects model.

Random effects	Name	Variance	SD	
items	Intercept	0.0006	0.0252	
participants	Intercept	0.0015	0.0386	
Residual		0.0054	0.0737	
Fixed effects	Estimate	SE	*t*-value	*p*-value
Intercept	2.785	0.0091	305.04	<0.001
perspective-first person	−0.0049	0.0019	−2.19	0.03
perspective-second person	0.0044	0.0019	2.28	0.02
context-fast	0.0028	0.0027	1.02	0.31
sentence plausibility	0.0021	0.0035	0.60	0.55
perspective-first person: context-fast	0.0061	0.0019	3.13	0.00
perspective-second person: context-fast	−0.0032	0.0019	−1.69	0.09

The facilitated verb recognition in the first-person perspective fast action sentences compared with that in the first-person perspective slow action sentences seemed to be consistent with previous research which found that action simulation can be modulated by semantic context (e.g., Moody and Gennari, 2010; Bidet-Ildei et al., 2017a; Sieksmeyer et al., 2021). One possible explanation for this might be that the fast action sentences were more semantically plausible and familiar than the slow action sentences. It is possible that the more plausible fast action sentences had a greater priming effect on the following verb processing due to the higher possibility of the co-occurrence of the verbs and other components of the sentences. While the less plausible slow action sentences had a higher processing load since the unfamiliar linguistic material needed more attention than familiar ones (e.g., Bicknell et al., 2010), thus interfering with the following verb recognition. However, cautions must be made to this explanation since the main effect of *sentence plausibility* was not observed (*x^2^*(1) = 6.54, *p* = 0.55), indicating that the differences in verb recognition between fast action sentences and slow action sentences may not be solely attributable to the semantic plausibility.

One more plausible explanation is that the activation of action representation was decreased when verbs were embedded in the slow action sentences. Although the target verbs emphasizing fast movement lead to fast action simulation in default, the slow action context cannot efficiently evoke or even coerce the fast-speed action simulation, thus slowing down the target verb processing. While the fast action context can enhance the fast action simulation, resulting in the fast recognition of the target verbs. To confirm this assumption, the 30 participants were asked to rate on a 7-point questionnaire about whether they could imagine themselves producing the actions described in the sentences. It has been found that the first-person perspective fast action sentences (*M* = 5.42, SD = 0.09) had a higher score on imaginability than the first-person perspective slow action sentences (*M* = 4.14, SD = 0.14) (*t* = 6.71, *p* < 0.001), which supports the hypothesis that fast action sentences are more likely to evoke action simulation.

In the fast action condition, the facilitated recognition of verbs following first-person perspective sentences compared with those following second-person or third-person perspective sentences matched results obtained in previous studies (e.g., Brunye et al., 2009; Ditman et al., 2010; van Dam et al., 2017), indicating that the mental simulation can be modulated by the perspective taking. It has been argued that in the first-person perspective context, the representational content is grounded more strongly based on the motor simulations, while in the third-person perspective context, the grounding of the representational content relies more on the visual simulation (van Dam et al., 2017). Following this hypothesis, the first-person perspective sentences in this experiment might evoke strong motor simulation in which the fine-grained speed information was included, facilitating the recognition of verbs. On the contrary, the third-person perspective sentences may rely more on perceptual simulation and less on motor simulation, thus cannot facilitate verb processing. However, Brunye et al. (2009) showed that the pronoun *you* and *I* elicited a similar mental simulation. This differs from the findings presented here. This intriguing finding might be explained by the fact that we have been explicitly instructed about the difference between first-person and second-person perspectives, and the first-person pronouns were more likely to elicit internal perspectives, thus leading in greater motor simulation.

It is somewhat surprising that the perspective seemed to have no effect on the verb recognition in the slow action condition. This may be explained in part by the trade-off between the coercion of the speed simulation in the first-person perspective condition and the weak motor simulation elicited in the second-person or third-person perspective conditions. As discussed earlier, the slow action sentences may coerce the simulation of fast action implied by the verbs. And the first-person perspective may further deepen this coercion. In addition, the perceptual-simulation-dependent third-person perspective sentences would lead to weak motion simulations. Therefore, the verb recognition in the first-person perspective slow action sentence did not significantly differ from that in the second- or third-person perspective slow context.

### 4.1 Experiment 3

Experiment 2 provided some tentative evidence that speed simulation could be modulated by the context even though the speed information is specified in the action representation. However, this account must be approached with some caution because the influence of sentence plausibility might not be completely excluded. Given this, in Experiment 3, we controlled the plausibility of sentences describing fast and slow movements and re-examined the influence of context on the speed simulation in neutral verb processing. Since verbs like *walk* do not convey specific speed properties, comprehending these verbs may lead to vague speed simulation. If the context is a modulator, then one may expect different responses to the neutral verbs embedded in different contexts. On the contrary, if the context exerts no effect, the speed simulation should be neutral, which may lead to a similar response to verbs in different sentences.

### 4.2 Method

#### 4.2.1 Participants

The 30 students participating in Experiment 2 also took part in Experiment 3.

#### 4.2.2 Materials

15 verbs depicting neutral speed movement were used as target verbs (e.g., “行走,” to walk). These verbs were rated in terms of speed by 20 raters who took part in Experiment 1, with the mean rating score being 3.567 (SD = 0.26). Similar to Experiment 2, each verb was used as the final word to create two kinds of sentences describing either a neutral speed (e.g., “在草地上行走,” to walk on the grass) or a relatively slow event by changing the prepositional-phrases preceded (e.g., “在沙漠中行走,” to walk in the desert). In addition, both the neutral speed sentences and the slow speed sentences were depicted from three different perspectives by changing the pronouns *I*, *you*, and *He/she.* Therefore, a total of 90 critical sentences can be assigned to six experimental conditions (15 sentences per condition).

Each sentence was rated in terms of the speed of the action depicted and the semantic plausibility by 20 participants who did not participate in the formal experiment on a 7-point scale questionnaire (1 being very slow and totally implausible, 7 being very fast and completely plausible). The speed rating of neutral speed sentences (*M* = 3.13, SD = 0.27) was significantly different from slow speed sentences (*M* = 2.85, SD = 0.34), *t* = 3.29, *p* = 0.01. But the neutral speed sentences (*M* = 5.47, SD = 0.27) were not more semantically plausible than slow speed sentences (*M* = 5.31, SD = 0.26) (*t* = 1.54, *p* = 0.15). Besides, the complements in neutral speed sentences and slow speed sentences were matched in terms of strokes (*mean neutral speed sentences* = 15.65, *mean slow speed sentences* = 16.53; *t* = −0.51, *p* = 0.62) and frequency (*mean neutral speed sentences* = 3821.29, *mean slow speed sentences* = 2122.82; *t* = 1.70, *p* = 0.11). In addition, 90 filler sentences were created by changing the final words of each sentence to pseudowords.

#### 4.2.3 Procedure

The procedure of Experiment 3 was the same as that of Experiment 2.

### 4.3 Data analysis

The data of one participant was excluded due to technical problems. For the rest data, the exclusion criteria were the same as that in Experiment 2 with about 2.7% trials being rejected. The results across conditions are summarized in Table 7. Similar to Experiment 2, the linear mixed modeling was employed to analyze the log-transformed reaction times. The fixed-effect predictors included the *context* (neutral speed vs. slow speed) and *perspective* (first-person vs. second-person vs. third-person). The crossed-random factors were participants and items.

**Table 7:** Average reaction times (RTs) with standard derivations (SDs) in Experiment 3.

	Neutral-peed (ms)	Slow-speed (ms)
First-person perspective	601 ± 125	623 ± 140
Second-person perspective	609 ± 134	626 ± 140
Third-person perspective	610 ± 135	625 ± 138

## 5 Results and discussion

Results showed that verb recognition was significantly influenced by the *context* (*x^2^*(1) = 5.17, *p* = 0.02), while the *perspective* and the interaction between *context* and the *perspective* exerted little influence on the verb processing (*ps*>0.05). Detailed analysis showed verb recognition following neutral speed sentences were significantly faster than that following slow speed sentences (*β* = −0.01, SE = 0.01, *t* = −2.26, *p* = 0.04).

The slower verb recognition in the slow speed condition indicated again that the context can modulate the simulation speed. This result was somewhat in agreement with previous studies indicating that the action simulation in verb processing is flexible (Bidet-Ildei et al., 2017b; Sieksmeyer et al., 2021). And this result might be attributed to the fact that the originally less certain speed information implied in verbs can be specified by the context. Since the speed information implied in the target verbs (e.g., 行走, to walk) was not specified, that is to say, neither fast nor slow, the simulation of the speed should have been neutral. However, the slow speed sentences imposed information of slow movement on the verbs and encouraged a relatively slow action simulation, thus resulting in slower responses. In other words, the verbs were gifted with specific speed information by the preceding context thus the speed simulation can be specified and modulated.

An alternative explanation for the different speed simulations in different contexts may be due to participants’ motor experiences. It has been reported that experience can influence mental simulations (Bidet-Ildei et al., 2010; Lyons et al., 2010). The more experience one has, the more likely the mental simulation occurs. For example, in the study carried out by Beauprez et al. (2020), participants were asked to decide whether the target action verb was consistent with the preceding picture. It has been found that action verb processing was facilitated when an action was perceived in its usual context, which demonstrated the role of motor experience in action verb processing. In experiment 3, the words used as complements were not controlled for the motor experience. That is to say, participants may have more experience concerning the event depicted in the neutral speed sentences (e.g., to walk on the platform) than that depicted in the relatively slow-speed sentences (e.g., to walk in the carriage). Therefore, the action simulation may be more likely to be elicited in the neutral speed context, which then facilitated the verb processing.

It is somewhat surprising that perspective does not appear to affect the simulation of speed information, nor does it modulate the influence of context on speed simulation. These findings are contrary to previous studies which have suggested that embodied perspective-taking plays an important role in action language processing (e.g., Sato and Bergen, 2013; van Dam and Desai, 2016). In addition, this outcome is also contrary to our findings in Experiment 2, which unveiled that in fast action sentence, perspective-taking modulated the verb processing. However, these results seem to be consistent with other research which found that the simulation of perspective is not universal for language processing (e.g., Brunye et al., 2009; Brunyé et al., 2016; Hartung et al., 2017). For instance, in a study by Hartung et al. (2017), participants were presented with stories containing either 1st or 3rd person pronouns referring to the protagonist while undergoing fMRI. The researchers found no evidence for neural dissociation depending on the pronoun when comparing action events. Besides, Brunyé et al. (2016) suggested that the patterns of internalization or externalization in response to the pronoun “I” can be influenced by both the context of the discourse and individual differences in empathic engagement.

One explanation of this intriguing finding is that the perspective information implied by pronouns may not be activated and simulated, hence not affecting the following verb processing. However, caution must be applied to this interpretation, since more studies, including the findings from our Experiment 2, have demonstrated that perspective information is subject to embodied simulation during language processing. Another explanation is that the simulation of perspective information is not necessary, or it may be conditional. In this study, participants were asked to make lexical decisions on the verbs following the prime sentences. Moreover, participants in Experiment 3 had all taken part in Experiment 2, which used the same paradigm. This could lead participants to rely more on linguistic cues rather than embodied simulation when making judgments (Louwerse and Jeuniaux, 2008). Therefore, the simulation of perspective information may be weak, and insufficient to influence the simulation of speed information in verb processing.

## 6 General discussion

A wealth of empirical evidence has shown language processing is embodied, and the mental simulation in the service of language comprehension is not schematic. The present study set out to explore whether detailed information like speed is included in the mental simulation, to investigate the conditions under which the speed simulation occurs, and to assess the flexibility of the fine-grained simulation of speed.

Experiment 1 generally confirmed that action simulations in action verb comprehension are detailed enough and contain information about motion speed. However, different responses to fast verbs and slow verbs were not significantly observed in the lexical decision task (Experiment 1a) but were significantly shown in the semantic similarity judgment task (Experiment 1b), which may indicate that the simulation of speed is more likely to occur on demand. In other words, the intensity of speed simulation would be modulated by task. Since the semantic similarity judgment could be considered as requiring a deeper level of semantic processing than the lexical decision, the speed information can be simulated in depth, making the difference between fast verbs and slow verbs more striking. Nevertheless, it can be inferred from these findings that action simulations of speed are an important component in the comprehension of verbs about speed.

Another significant finding to emerge from this study is that the simulation of speed in verb comprehension can be modulated by the context. Both Experiment 2 and Experiment 3 found that the recognition of verbs embedded in sentences depicting relatively slow action events would be slowed down, even when the sentence plausibility was controlled (see Experiment 3). If the simulation of speed in verb processing is invariant or is done in the default way, the co-occurring concepts in the sentences would not affect this action simulation and accordingly exert no effect on the verb processing. The slowing down verb processing just proved that the action simulation of speed was influenced by other components of the sentence (e.g., the prepositional phrases). It is worth noting that the primary difference between Experiments 2 and 3 lies in the target verbs. The speed information implied in the verbs used in Experiment 2 is quite precise regardless of context whereas verbs used in Experiment 3 may be rather vague. However, similar modulating effects of context on the recognition of verbs were observed. Therefore, it is reasonable to think that on the one hand, context can force a change in the nature of the described action, resulting in a “context coercion” that inhibits the original simulation of fast action. On the other hand, context can specify the vague aspects of the described action, resulting in a “context specification” that regulates the original simulation of neutral-speed action. However, the modulating effect of perspectives on verb processing was found in Experiment 2 but not in Experiment 3. It remains unclear whether the “context coercion” and “context specification” of speed simulation can be enhanced by perspective-taking.

There are possible limitations to the present study. Firstly, our study may be limited by the relatively small number of participants. Future research will involve extended samples to further validate the flexible and fine-grained simulation of speed in language processing. Secondly, although items were matched on various linguistic variables, it is possible that other relevant variables, such as age of acquisition and number of orthographic neighbors, could influence reaction times. In future research, we will implement more rigorous control over potential variables to enhance the reliability and validity of our findings. Thirdly, the effector variable was not taken into account in Experiments 2 and 3. It is argued that different effectors may elicit different levels of sensorimotor representations, resulting in different embodied simulations. Future studies will further consider the impact of the effector variable on speed simulation.

In conclusion, this study provides further evidence for the simulation of action speed, suggesting that simulations are not reconstructing action events in a schematic and coarse way, but include fine-grained information about the manner of action. However, the simulation of action speed is not so automatic in action language processing. In addition, we provide evidence that speed simulation is flexible which can be coerced or specified by the context.

## Data availability statement

The original contributions presented in the study are included in the article/Supplementary material, further inquiries can be directed to the corresponding author.

## Ethics statement

The studies involving humans were approved by the studies involving human participants were reviewed and approved by Anhui Xinhua University. The studies were conducted in accordance with the local legislation and institutional requirements. The participants provided their written informed consent to participate in this study.

## Author contributions

XP: Conceptualization, Data curation, Formal analysis, Funding acquisition, Methodology, Project administration, Resources, Software, Validation, Visualization, Writing – original draft, Writing – review & editing. BL: Data curation, Methodology, Project administration, Resources, Writing – review & editing. XL: Data curation, Investigation, Project administration, Supervision, Validation, Visualization, Writing – review & editing.
