# Supplementary material for: Flexible and fine-grained simulation of speed in language processing
Source: Front Psychol. 2024 Apr 10;15:1333598. doi: 10.3389/fpsyg.2024.1333598 (PMC11040083; doi:10.3389/fpsyg.2024.1333598)
Supplement: Supplementary file 1 [file Table_1.DOCX]

**Table S1** Verbs and Mean Speed Ratings

| Verb | Mean speed rating |  | Verb | Mean speed rating |
| --- | --- | --- | --- | --- |
| *Fast-full body* | |  | *Fast-hand* | |
| \| 奔跑 \| \| --- \| \| 快跑 \| \| 奔逐 \| \| 奔走 \| \| 快步 \| \| 疾走 \| \| 飞跑 \| \| 疾步 \| \| 小跑 \| \| 跑步 \| \| 奔逃 \| \| 追赶 \| \| 逃窜 \| \| 蹦跳 \| \| 闯入 \| \| 蹿动 \| | 5.2  5.4  5.45  4.45  4.45  5.1  5.9  5.1  4.25  4.35  5.55  5.2  5.25  4.35  4.9  4.35 |  | \| 捶打 \| \| --- \| \| 敲击 \| \| 鞭打 \| \| 掌嘴 \| \| 拍打 \| \| 抢夺 \| \| 掀开 \| \| 摔打 \| \| 鼓掌 \| \| 抛洒 \| \| 甩开 \| \| 投掷 \| \| 狂甩 \| \| 猛打 \| \| 捅入 \| \| 抽打 \| | 5  4.15  4.8  4.25  4.15  5.15  4.15  4.6  4.15  4.2  4.5  4.25  5.15  5.4  5.1  4.7 |
| *Slow-full body* | |  | *Slow-hand* |  |
| \| 挪步 \| \| --- \| \| 踱步 \| \| 缓步 \| \| 款步 \| \| 缓行 \| \| 漫步 \| \| 慢行 \| \| 徐步 \| \| 散步 \| \| 闲步 \| \| 徐行 \| \| 溜达 \| \| 逛游 \| \| 信步 \| \| 蹒跚 \| \| 跛行 \| | 2.55  2.55  2.6  2.65  2.9  2.95  2.9  3.15  3  2.75  3  3.05  3.35  3.2  2.1  2.65 |  | \| 抚摸 \| \| --- \| \| 拖动 \| \| 扛起 \| \| 托举 \| \| 摸索 \| \| 移开 \| \| 拗断 \| \| 挪移 \| \| 揉搓 \| \| 轻放 \| \| 捧起 \| \| 摆放 \| \| 扶起 \| \| 挽起 \| \| 轻拍 \| \| 轻拿 \| | 2.15  2.7  3.4  3.25  2.55  2.95  3  3.3  2.7  2.3  2.6  2.6  2.55  2.65  2.6  2.35 |

**Table S2** Experimental sentences used in Experiment 2

| Sentence | Mean speed rating |  | Sentence | Mean speed rating |
| --- | --- | --- | --- | --- |
| *Fast action sentence* | |  | *Slow action sentence* | |
| 在操场上飞奔  (Running in the playground)  在草地上奔跑  (Running on the grass)  在赛道上狂奔  (Running wild on the track)  在走廊里奔逐  (Chasing in the corridor)  在村子里奔走  (Running through the village)  在院落里追赶  (Chasing in the courtyard)  在大道上追逐  (Chasing on the avenue)  在原野上疾驰  (Speeding through the wilderness)  在马路上飞驰  (Speeding on the road)  在草场上奔驰  (Running on the meadow)  在暖阳里奔窜 (Running in the warm sun)  在平路上小跑 (Trotting on the flat road)  在牧场上快跑 (Running fast in the pasture)  在草丛里逃窜  (Scampering through the grass)  在跑道上赛跑  (Racing on the track)  在球场里跑步  (Running in the field)  在长廊里奔逃  (Running away in the corridor)  在晨光中疾行  (Sprinting in the morning light)  在站台上疾走 (Sprinting on the platform)  在花丛中飞跑  (Running through the flowers) | 5.8  5.05  6.4  4  4  4.45  5  6.3  6  5.75  4.85  4.05  5.4  4.8  5.85  4.8  4.95  4.5  4.1  4.8 |  | 在陡坡上飞奔  (Running on the steep slopes)  在雪地上奔跑  (Running on the snow)  在沙丘上狂奔  (Running wild on the sand dunes)  在丛林里奔逐  (Chasing through the jungle)  在淤泥里奔走  (Running in the mud)  在地道里追赶  (Chasing through the tunnels)  在教室里追逐  (Chasing in the classroom)  在山路上疾驰  (Speeding down the mountain path)  在石滩上飞驰  (Speeding on rocky beaches)  在积雪上奔驰  (Running on the snow)  在浓雾里奔窜  (Running through the fog)  在吊桥上小跑  (Trotting on the suspension bridge)  在山崖上快跑  (Running on the cliffs)  在废墟里逃窜 (Running through the ruins)  在沙滩上赛跑  (Racing on the beach)  在车厢里跑步  (Running in a carriage)  在洞穴里奔逃  (Running through caves)  在夜幕中疾行  (Racing through the night)  在水沟里疾走  (Sprinting through ditches)  在泥潭中飞跑  (Running through mud puddles) | 4.55  4.15  4.05  4.55  3.1  3.85  4.15  4.65  4.25  4.3  4.35  4.15  4.6  4.3  4.7  3.35  4  4.2  3.35  3.9 |

**Table S3** Experimental sentences used in Experiment 3

| Sentence | Mean speed rating |
| --- | --- |
| *Neutral speed sentence* |  |
| 在草地上行走  (Walking on the grass)  在站台上走动  (Walking on the platform)  在和风中步行  (Walking in the gentle breeze)  在晨光中前行  (Walking in the morning light)  在马路上行进  (Walking on the road)  在花丛中穿行  (Walking through the flowers)  在草丛中迈步  (Striding through the grass)  在夕阳中巡行  (Cruising in the sunset)  在平原上徒步  (Hiking on the plain)  在晚霞中前进  (Advancing in the evening sun)  在大道上走路  (Walking on the avenue)  在微风中行路  (Walking in the breeze)  在展厅间来回 (Back and forth between showrooms)  在平地上后退  (Retreating on the flat)  在石桥上移动  (Moving on the stone bridge) | 3.3  2.85  2.85  3  3.4  3.5  3.35  3.35  2.95  3.4  3.35  3.1  3.2  2.7  2.7 |
| *Slow speed sentence* | |
| 在暴雨中行走  (Walking in the rainstorm)  在车厢中走动  (Walking in the carriage)  在风沙中步行  (Walking in the sand and wind)  在浓雾中前行  (Walking in the fog)  在雨林中行进  (Walking in the rainforest)  在迷宫中穿行  (Walking through the maze)  在泥潭中迈步  (Striding through mud puddles)  在夜幕中巡行  (Cruising through the night)  在沙漠中徒步  (Hiking in the desert)  在雾气中前进  (Advancing through the fog)  在地道中走路  (Walking in the tunnels)  在疾风中行路  (Walking in the wind)  在石堆间来回  (Back and forth between piles of rocks)  在泥水中后退  (Retreating in the mud and water)  在吊桥上移动  (Moving on the suspension bridge) | 3.5  3.1  2.6  2.65  3.15  3.05  2.25  2.95  2.75  2.8  3.2  2.6  3.15  2.4  2.65 |
